# Atypical hemolytic‐uremic syndrome after COVID‐19 vaccine: A case report

**DOI:** 10.1002/iid3.1270

**Published:** 2024-07-05

**Authors:** Marcos Adriano Garcia Campos, Rômullo José Costa Ataídes, Maxwell Cabral Ferreira, Adriano Soares Alves, Gyl Eanes Barros Silva

**Affiliations:** ^1^ Clinical Hospital of Botucatu Medical School of São Paulo State University Professor Mário Rubens Guimarães Montenegro Avenue Botucatu Brazil; ^2^ Faculty of Medicine of Federal University of Maranhão Gonçalves Dias Square São Luís Brazil; ^3^ Faculty of Medicine of University Center of Maranhão São Luís Brazil; ^4^ Dr Carlos Macieira Hospital Jerônimo de Albuquerque Avenue São Luís Brazil

**Keywords:** atypical hemolytic‐uremic syndrome, case report, post‐COVID‐19, thrombotic microangiopathy

## Abstract

**Background:**

The emergence of new SARS‐CoV‐2 variants and the global COVID‐19 pandemic spurred urgent vaccine development. While common vaccine side effects are well‐documented, rare adverse events necessitate post‐marketing surveillance. Recent research linked messenger RNA vaccines to thrombotic microangiopathy (TMA), a group of syndromes characterized by microvascular hemolytic anemia and thrombocytopenia. This report describes a new‐onset atypical hemolytic‐uremic syndrome (aHUS) occurring after COVID‐19 vaccination and complements recent literature.

**Case Presentation:**

A previously healthy 25‐year‐old woman developed malaise, nausea, edema, and renal dysfunction 60 days postvaccination. Laboratory findings confirmed TMA diagnosis. Genetic testing for complement system mutations was negative. Kidney biopsy supported the diagnosis, and the patient required hemodialysis.

**Conclusion:**

This case illustrates the rare occurrence of aHUS following COVID‐19 vaccination, with unique characteristics compared to previous reports. Despite the critical role of vaccination in pandemic control, emerging adverse events, such as vaccine‐related TMA, must be recognized and investigated. Additional clinical trials are imperative to comprehend the clinical features and pathophysiological mechanisms underlying TMA associated with COVID‐19 vaccination.

## Graphical abstract

The purpose of this report is to describe a new‐onset atypical hemolytic‐uremic syndrome that occurred after the administration of the COVID‐19 vaccine.

## 1 INTRODUCTION

With the emergence of new variants of the SARS‐CoV‐2 coronavirus and the continued spread of COVID‐19, the urgent need for vaccine production arose.^1^ The side effects of each vaccine are well‐documented and published at the time of licensing. Despite the most frequent side effects of COVID‐19 vaccines, uncommon vaccine‐related adverse events have been documented and necessitate postmarketing monitoring.^2^ A recent review of 84 new‐onset or relapsed thrombotic microangiopathy (TMA) cases post‐COVID‐19 vaccination showed a strong association with messenger RNA vaccines.^3^ TMA refers to a group of syndromes characterized by microvascular hemolytic anemia and thrombocytopenia with a negative Coombs test and damage to target organs such as the heart, kidneys, lungs, and central nervous system.^4^ There are three main types of TMA: thrombotic thrombocytopenic purpura (TTP), hemolytic‐uremic syndrome (HUS), and atypical hemolytic‐uremic syndrome (aHUS). The latter consists of several subtypes that can be distinguished by their etiology, including pregnancy‐induced HUS and drug‐induced HUS.^5^, ^6^

The purpose of this report is to describe a new‐onset aHUS that occurred after the administration of the COVID‐19 vaccine and complement the most recent literature review.

## 2 CASE REPORT

A previously healthy 25‐year‐old woman, with a history of mild flu‐like symptoms due to documented COVID‐19 in April 2020, received the first dose of the ChAdOx1 nCov‐19 vaccine ‐ AstraZeneca on June 15, 2021. After 15 days, she began experiencing malaise, nausea, vomiting, abdominal distension, and asthenia.

Subsequently, she developed edema in the lower limbs, dyspnea with exertion, persistent abdominal pain, and nausea. The Patient was treated at the emergency department according to her symptoms and she was discharged after 10 days with improvement of complaints. No specific diagnosis was made. Creatinine and proteinuria values at discharge were not available.

After 60 days from vaccine administration, she presented to the emergency department with the symptoms above plus hypertension (190/140 mmHg). Admission laboratory tests revealed renal dysfunction ‐ blood urea nitrogen (BUN) 5.21 mmol/L and creatinine 4.58 mg/dL ‐ and hemoglobinuria.

Initially, there were no changes in the complete blood count. Differential diagnosis of edema and secondary hypertension was investigated. Echocardiography and BNP levels were normal, with a BNP of 28.51 pg/mL (normal < 100.00 pg/mL). After 5 days of supportive clinical treatment, the patient's clinical condition improved, and she was discharged with a prescription for Captopril 25 mg once daily and referred for outpatient follow‐up with the nephrology department for further investigation.

Three weeks after hospital discharge (86 days after vaccination), the patient was again brought to the emergency department, this time with acute hypertensive pulmonary edema. Initial admission tests showed worsened renal function (oliguria, BUN 14.6 mmol/L, creatinine 18.7 mg/dL), anemia (hemoglobin 10.1 g/dL), neutrophilic leukocytosis (20,980/mm³), and thrombocytopenia (143,000/mm³). Infectious diseases was excluded due low c‐reactive protein, negative blood and urine cultures, and no suggestive symptoms. Clinical improvement was achieved with prompt initiation of invasive ventilatory support, parenteral antihypertensive treatment, and renal replacement therapy.

Laboratory investigation was consistent with the diagnosis of TMA: positive schistocytes on the peripheral blood smear, elevated lactate dehydrogenase 886 U/L, negative direct Coombs test, proteinuria of 2.95 g/24 h, and urinalysis with important hemoglobinuria. Research into mutations in genes encoding complement system components was negative for ADAMST13, CD46, CFH, CFHR2, CFHR4, CFI, THBD, C3, CFB, CFHR1, CFHR3, CFHR3, CFHR5, and DGKE. The rest of the investigation was negative (blood culture, urine culture, serology for HIV, hepatitis B and C, syphilis, ANCA, ANA, anti‐DNA, complement levels, ADAMTS‐13 assay, and flow cytometry).

**Figure 1:**
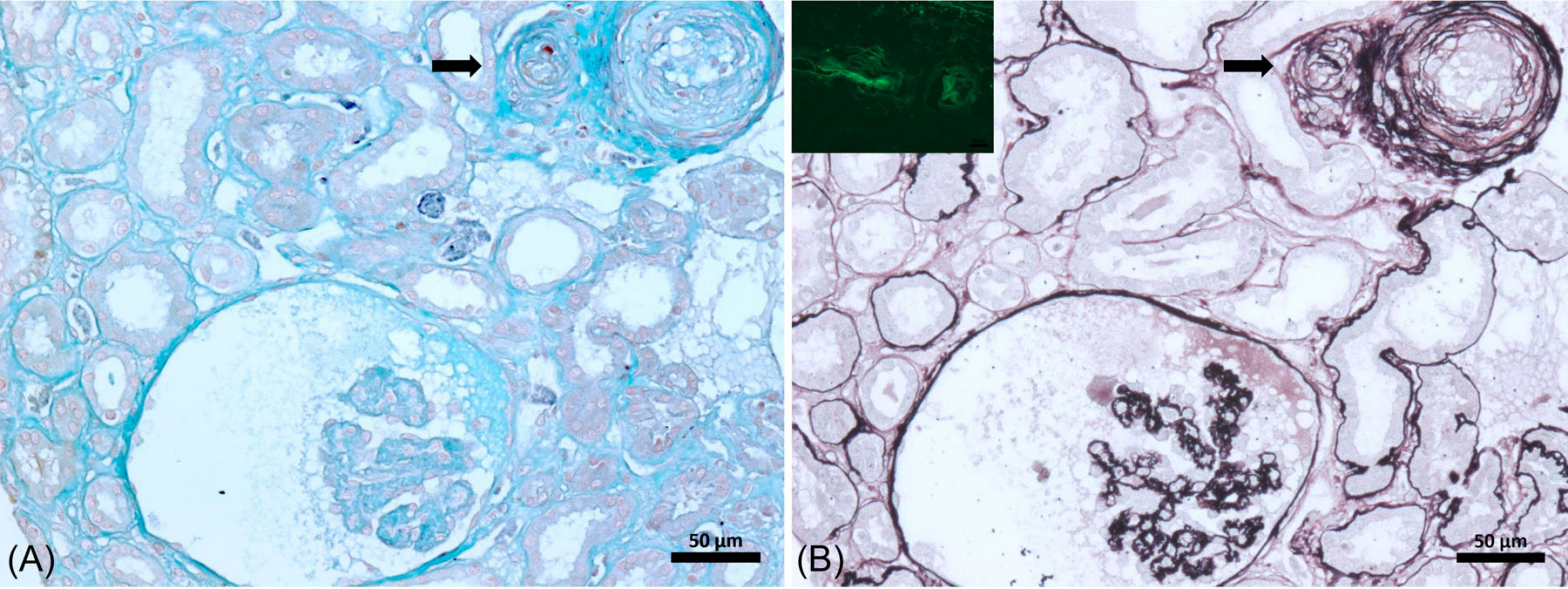
Arteries walls with concentric lamination, one of them with small fibrin thrombus (arrow). The adjacent glomerulus has global ischemic collapse with expanded mesangial regions and endothelial cell swelling (A) Masson's Trichrome; (B) Silver stain. Inset image: vessel wall shows moderate fibrinogen positivity by direct immunofluorescence.

The patient underwent a kidney biopsy, which revealed 12 glomeruli, one of which was globally sclerotic, and 11 showed marked ischemic changes, along with discrete areas of interstitial fibrosis, moderate to severe tubular degeneration, and moderate intimal thickening of vessels on light microscopy. Immunofluorescence showed all immunoglobulins negative, and only fibrinogen was positive on vessels. The renal biopsy was consistent with thrombotic microangiopathy with minimal tubulointerstitial involvement (10‐20%) and associated marked acute tubular necrosis (Figure 1).

After initiating renal replacement therapy, the patient's clinical condition improved with satisfactory blood pressure control, ventilatory weaning, subsequent extubation, and stabilization of nitrogenous waste products. No specific treatment for aHUS was performed. The main treatment was related to the management of end‐stage renal disease. The patient was discharged from the hospital after 2 weeks. Currently, the patient is receiving outpatient follow‐up care and continues to have urinary production; however, there remains inadequate clearance necessitating hemodialysis treatment. The timeline of events is described in Table 1.

**Table 1:** Timeline of events.

Date	Event
April 2020	The patient, a previously healthy 25‐year‐old woman, experienced mild flu‐like symptoms due to documented COVID‐19 infection.
June 15, 2021	The patient received the first dose of the ChAdOx1 nCov‐19 vaccine (AstraZeneca).
15 days postvaccination	The patient began experiencing symptoms, including malaise, nausea, vomiting, abdominal distension, and asthenia.
15 days postvaccination	The patient's condition worsened, and she developed edema in the lower limbs, dyspnea with exertion, persistent abdominal pain, and continued nausea.
15−60 days postvaccination	Patient was treated according to her symptoms and she was discharged after 10 days with improvement of complaints. No specific diagnosis was made.
60 days postvaccination	The patient presented to the emergency department with the symptoms mentioned above, along with hypertension (190/140 mmHg).
Admission laboratory tests (60 days postvaccination)	Laboratory tests revealed renal dysfunction, with blood urea nitrogen (BUN) at 94 mg/dL and creatinine at 4.58 mg/dL. Hemoglobinuria was also noted.
After 60 days postvaccination	There were no changes in the complete blood count, and the patient received supportive clinical treatment.
86 days postvaccination (three weeks after initial hospital discharge)	The patient was readmitted to the emergency department, this time with acute hypertensive pulmonary edema.
Admission tests (86 days postvaccination)	Tests showed worsened renal function with oliguria, BUN at 188 mg/dL, creatinine at 18.7 mg/dL, anemia (hemoglobin 10.1 g/dL), neutrophilic leukocytosis (20,980/mm³), and thrombocytopenia (143,000/mm³).
Treatment (86 days postvaccination)	Clinical improvement was achieved with prompt initiation of invasive ventilatory support, parenteral antihypertensive treatment, and renal replacement therapy.
Laboratory investigation (86 days postvaccination)	Laboratory investigation confirmed the diagnosis of thrombotic microangiopathy (TMA) with positive schistocytes on peripheral blood smear, elevated lactate dehydrogenase (886 U/L), negative direct Coombs test, proteinuria of 2.95 g/24 h, and urinalysis with significant hemoglobinuria.
Genetic testing (86 days postvaccination)	Research into mutations in genes encoding complement system components was negative for various factors.
Kidney biopsy (86 days postvaccination)	A kidney biopsy revealed characteristics consistent with TMA, including glomerular changes, interstitial fibrosis, tubular degeneration, and vessel thickening.
After kidney biopsy (86 days postvaccination)	Following the biopsy, renal replacement therapy was initiated, leading to clinical improvement.
Hospital discharge (approximately two weeks after readmission)	The patient was discharged from the hospital.
Current status	The patient is currently receiving outpatient follow‐up care, and while she continues to have urinary production, there remains inadequate clearance necessitating hemodialysis treatment.

## 3 DISCUSSION

Although vaccination campaigns have contributed to the decline in COVID‐19 rates, adverse events are emerging beyond those initially primarily in the efficacy and safety clinical trials of the vaccine. Relevant to the renal system, there is a growing number of reports of new cases or reactivation of glomerular diseases, such as IgA nephropathy‐related, minimal change disease with nephrotic syndrome, and ANCA‐associated vasculitis.^7^

The case we present is an example of aHUS that developed following vaccination against COVID‐19, as Aigner et al.^8^ documented. It is worth noting that thrombotic microangiopathy occurs in only approximately 5% of cases, according to these same authors.^8^ Furthermore, comprehensive evaluations have excluded primary factors such as paroxysmal nocturnal hemoglobinuria, thrombotic thrombocytopenic purpura, HUS, and malignant hypertension; as well as secondary causes including autoimmune disorders, pregnancy‐related conditions, infections, and medication use.^9^ On the face of that, our analysis proposes a linkage between this case and complement‐mediated thrombotic microangiopathy triggered by the administration of the ChAdOx1 nCov‐19 vaccine ‐ AstraZeneca. COVID‐19 vaccination can lead to a pro‐inflammatory state with disturbances in complementary activation and the coagulation cascade, which can cause TMA.^9^

In this case, distinguishing between TMA is essential. Signs of microvascular hemolytic anemia, thrombocytopenia, and acute kidney injury point towards TMA, while the absence of large vessel clots and normal d‐dimer levels rule out vaccine‐induced thrombotic thrombocytopenia (VITT). Atypical hemolytic uremic syndrome (aHUS) is more likely, supported by a negative Coombs test, schistocytes presence, and kidney biopsy findings indicative of microangiopathy. The connection of symptoms post‐COVID‐19 vaccination to aHUS, rather than VITT, emphasizes the need for ongoing monitoring and research into vaccine‐related complications.

The presented case shows similarity with the results for Ma Q and Xu G review,^3^ being more incident in women, after the first dose of immunization without renal alteration previously. However, it contrasts by presenting itself in a patient of age below the average age of the cases reported, in addition to having been caused after the application of an adenoviral vector vaccine (as shown in another study),^10^ starting symptoms after 15 days of the administration and not showing complete remission of the case and still requiring hemodialysis. However, even in patients already diagnosed with renal impairment, the incidence of aHUS after vaccination for COVID‐19 is low.^9^ A review of reported aHUS cases described in the literature is summarized in Table 2.

**Table 2:** Summary of reported literature cases about atypical hemolytic‐uremic syndrome after COVID‐19 vaccination.

Type of vaccine	Number of cases per dose	Average of onset time	Sex	Average of age
mRNA vaccine	First dose: 3	9.9 ± 11.8 days	Male: 6	34.0 ± 18.7 years
	Second dose: 6		Female: 4	
	Third dose: 1			
Viral vector vaccine	First dose: 5	3.7 ± 2.0 days	Male: 5	46.7 ± 14.4 years
	Second dose: 2		Female: 2	
	Third dose: 0			

*Note*: See references.^11^, ^12^, ^13^, ^14^, ^15^, ^16^, ^17^, ^18^, ^19^, ^20^, ^21^, ^22^, ^23^, ^24^

In conclusion, while the administration of COVID‐19 vaccines has played a crucial role in reducing case rates and tackling the global pandemic, it is vital to acknowledge the emerging adverse events associated with these vaccines, We agree on the need for more reports of clinical trials are needed to increase understanding of the clinical characteristics and mechanism of the physiopathology of TMA associated with COVID‐19 vaccination.

## AUTHOR CONTRIBUTIONS

**Marcos Adriano Garcia Campos**: Writing—original draft. **Rômullo José Costa Ataídes**: Writing—original draft. **Maxwell Cabral Ferreira**: Conceptualization; data curation. **Adriano Soares Alves**: Conceptualization; data curation. **Gyl Eanes Barros Silva**: Resources; supervision; writing—review and editing.

## CONFLICT OF INTEREST STATEMENT

The authors have no conflicts of interest to declare.

## Data Availability

The authors have nothing to report.
